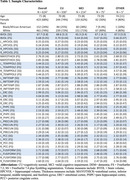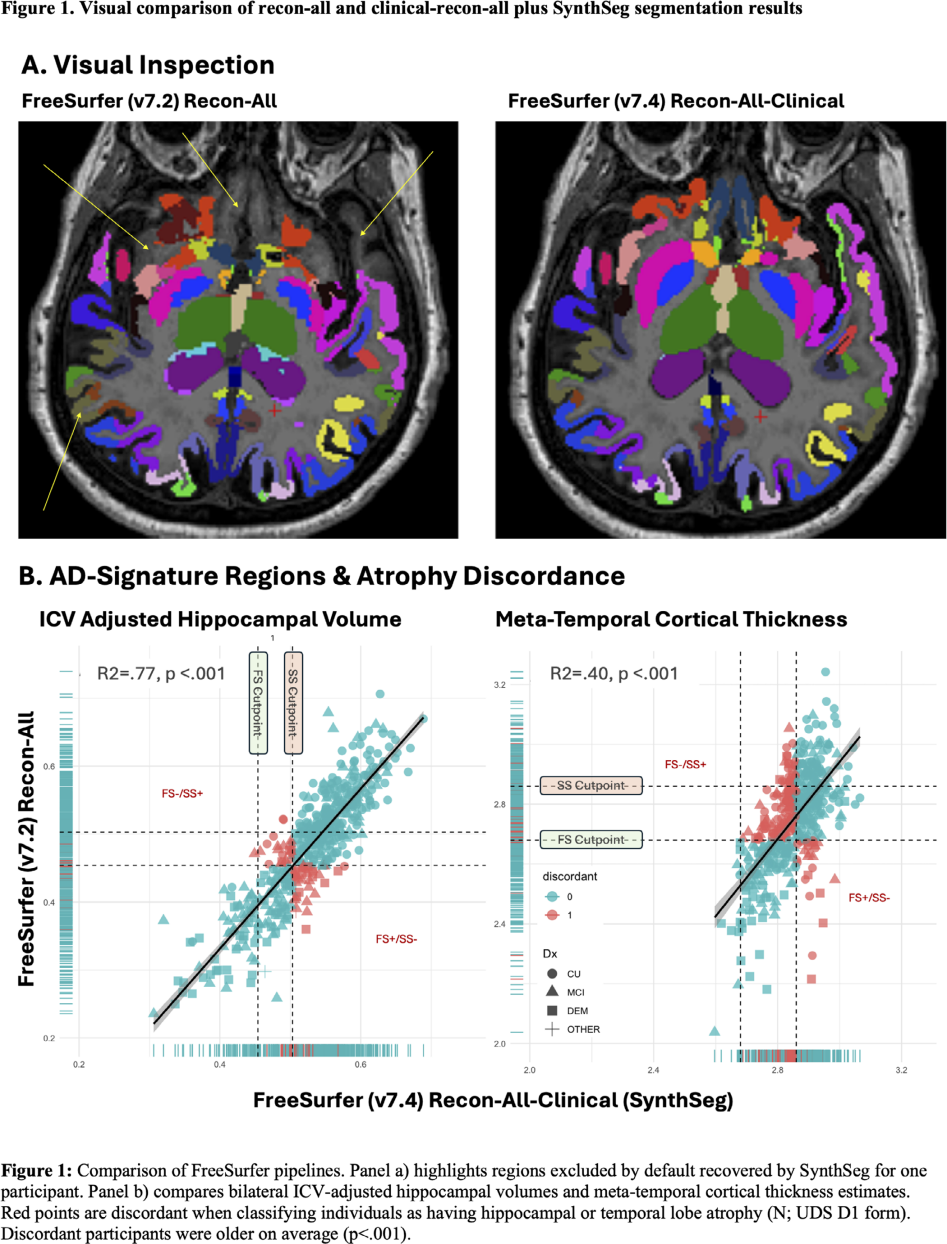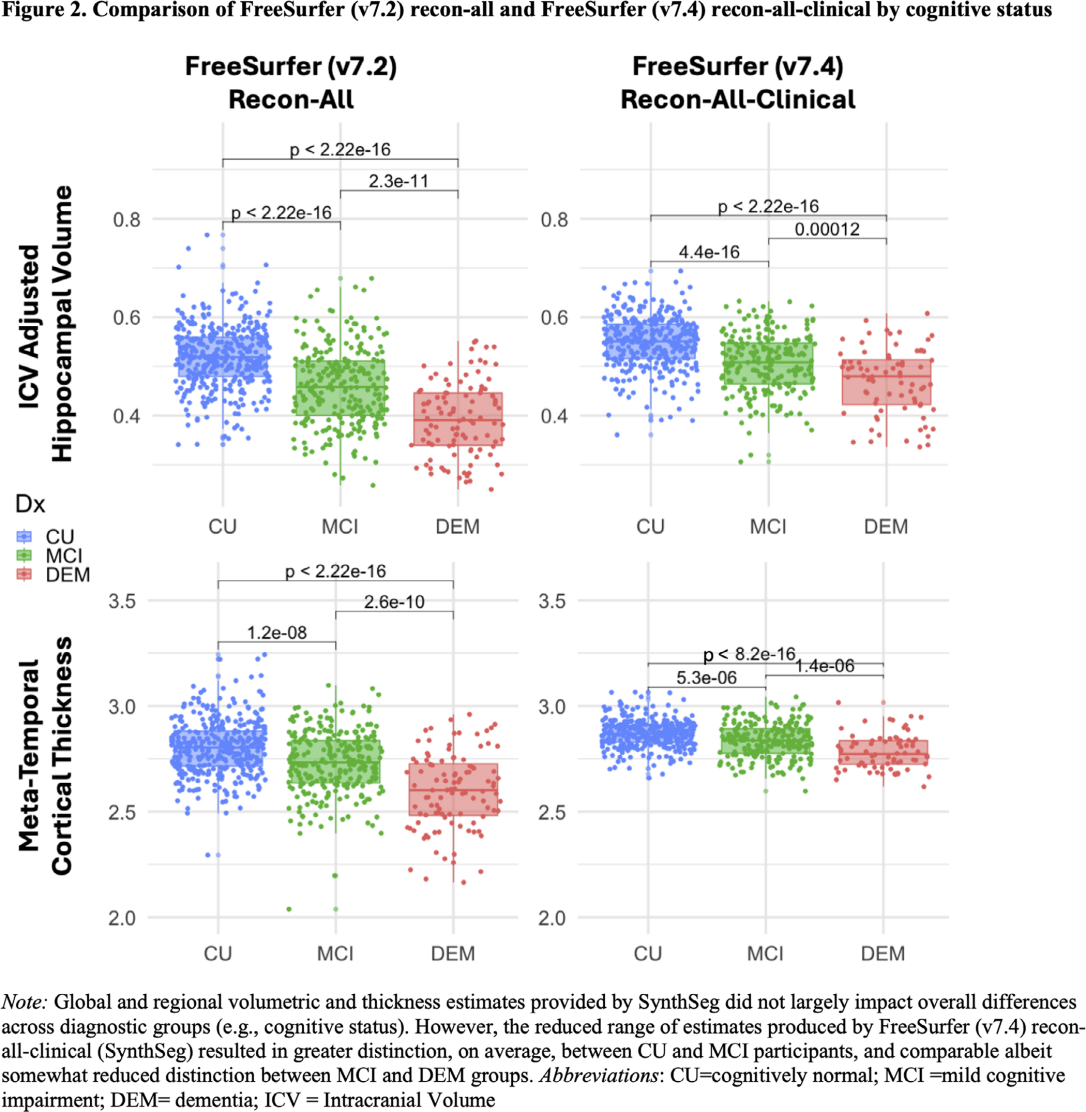# Preliminary comparison of FreeSurfer segmentation algorithms in the Wake Forest community‐based cohort and potential impact on ATN classification

**DOI:** 10.1002/alz70856_107027

**Published:** 2026-01-13

**Authors:** Marc D. Rudolph, Melissa M. Rundle, Kathryn H Alphin, Richard A. Barcus, Timothy M. Hughes, Trey R. Bateman, Kiran K. Solingapuram Sai, Christopher T Whitlow, Suzanne Craft, Da Ma

**Affiliations:** ^1^ Wake Forest University School of Medicine, Winston‐Salem, NC, USA; ^2^ Wake Forest School of Medicine, Winston‐Salem, NC, USA

## Abstract

**Background:**

Acquisition and participant‐related artifacts (atrophy, motion) can degrade the quality of acquired images resulting in poor segmentation of tissue compartments. This can bias estimates of brain volume and thickness used quantify age and disease‐related atrophy, a problem particularly salient in clinical populations. In some cases, poor quality scans may be discarded or repeated incurring additional costs.

**Method:**

Participants (*n* = 624; cognitively normal [CU;*n* = 330]; mild cognitive impairment [MCI;*n* = 214]; dementia [DEM;*n* = 75]; otherwise not classified [OTHER;*n* = 5)] enrolled in the Wake Forest ADRC Clinical Cohort (Table 1). Structural T1‐MRI scans were processed using FreeSurfer (v7.2) recon‐all and FreeSurfer (v7.4) recon‐all‐clinical (SynthSeg) pipelines. Tau‐PET (FTP) images were acquired; global, meta‐temporal, and entorhinal tau‐PET (white+gray matter; SUVr) was quantified. Measures of (1) cortical thickness: entorhinal cortex, inferior temporal lobe, temporal pole, and meta‐temporal; (2) volume: gray matter, white matter, and hippocampi; and (3) tau deposition (global and entorhinal SUVr) were compared between pipelines and by cognitive status (Figure 2). GLMs (R^2^=shared variance) and gaussian‐mixture modeling (cohort‐specific atrophy cutpoints) were performed.

**Result:**

Overall, FreeSurfer (v7.2) recon‐all tended to undersgement, producing smaller volume and thickness estimates (and a wider range of estimates), as compared to FreeSurfer recon‐all‐clinical (e.g., SynthSeg; Figure 1a). For cortical thickness, we observed poor‐to‐moderate associations in signature regions for age‐related dementias including: temporal pole [R^2^: Left=3%; Right=7%]); inferior temporal lobe (R^2^: Left=34%; Right=24%), entorhinal cortex (R2: Left=32%; Right=27%), posterior cingulate (R^2^: Left=40%; Right=39%), and precuneus (R^2^: Left=39%; Right=36%). Conversely, volumetric estimates were largely comparable across pipelines, except for hippocampi (R^2^: Left=65%; Right=64%), where we observed a modest drop in agreement for classification of atrophy (N; Figure 1b). ∼13% (hippocampal volume) and 36% (meta‐temporal cortical thickness) of cases were discordant when classifying atrophy (Figure 2: cognitive status). Quantification of tau‐PET (global/regional deposition) was not impacted.

**Conclusion:**

Volumetric estimates were comparable across FreeSurfer segmentation algorithms in our cohort; however, cortical thickness estimates were impacted contributing to discrepant classification of atrophy. SynthSeg segmentations were robust to scan quality (not shown) and recovered susceptible regions (e.g., temporal pole). Deep learning‐based segmentation algorithms (e.g., SynthSeg) require less processing time and may ultimately reduce the need to discard poor quality scans or perform manual segmentation.